# Developmental plasticity in response to early-life conditions affects male sexual traits in guppies

**DOI:** 10.1093/beheco/arag096

**Published:** 2026-08-19

**Authors:** Diego Moura-Campos, Meng-Han Joseph Chung, Megan L Head

**Affiliations:** Research School of Biology, Division of Ecology and Evolution, Australian National University, 46 Sullivans Creek Road, Acton, ACT 2601, Australia; Research School of Biology, Division of Ecology and Evolution, Australian National University, 46 Sullivans Creek Road, Acton, ACT 2601, Australia; Centre for Conservation Ecology and Genomics, Institute for Applied Ecology, University of Canberra, University Drive, Bruce, ACT, Australia; Research School of Biology, Division of Ecology and Evolution, Australian National University, 46 Sullivans Creek Road, Acton, ACT 2601, Australia

**Keywords:** compensatory growth, catch-up growth, reproduction, climate change, poeciliid, sperm, sexual selection, male–male competition, sexual display, telomere

## Abstract

Early environmental conditions play an important role in animal development. Lack of sufficient food early in life can lead to impaired growth and long-term fitness consequences. To avoid that, many animal species compensate during a period of low food availability by increasing their growth rate when food becomes abundant again, ie compensatory growth. However, compensatory growth during development may divert resources away from the ontogeny of reproductive and somatic functions, imposing hidden costs that are only apparent later in life. For ectotherms, these trade-offs are expected to be exacerbated by increased temperatures due to the high energetic demands of increased metabolism. We test if diet restriction during early development has long-term effects on the reproductive traits of male Trinidadian guppies (*Poecilia reticulata*), and, if so, whether such effects are exacerbated in warmer temperatures. Previously, we have shown that diet-restricted guppies exhibit compensatory growth. Here, we found that diet restriction during early development had no effect on the expression of sexual traits later in life. In contrast, males experiencing elevated temperatures throughout life exhibited a reduction in both black body coloration and dominance behaviors and died younger. Surprisingly, temperature did not mediate the effects of early-life diet on male sexual traits. However, temperature did mediate the effects of diet on adult growth, with diet-restricted males showing impaired growth later in life but only when they experienced elevated temperatures. Our results highlight the developmental plasticity of sexual traits and suggest that future climate predictions have the potential to negatively affect fish reproduction.

## Teaser

We tested whether, in guppies, male reproduction was affected by compensatory growth following diet restriction during early development. We also tested whether these effects varied under different temperatures. Surprisingly, compensatory growth after diet restriction had no effects on any reproductive traits, apart from a reduction in adult size under elevated temperatures. Elevated temperatures independently reduced male dominance, black coloration and survival. Our findings show no long-term costs of compensatory growth on male secondary sexual traits, even under warmer conditions.

## Introduction

Environmental conditions can change faster than organisms can adapt to them. For that reason, many organisms have evolved to express different phenotypes from the same genotype (Via and Lande 1985; Schlichting and Pigliucci 1998). This phenotypic plasticity can allow animals to develop traits that match environmental conditions and, therefore, maintain their overall fitness (Bradshaw 1965; Scheiner 1993; Miner et al. 2005). For animals, one of the most critical and limiting conditions they can face during development is fluctuating food availability. Predation pressure, population density, and fluctuations in the abundance of prey can all alter levels of food availability. If such environmental variation is experienced early in life (ie during development), it may lead to impaired growth, altered metabolism, and increased mortality (Eyck et al. 2019; Almeida et al. 2021). To avoid possible fitness costs associated with reduced food intake during development, animals have evolved a variety of strategies to cope with short-term changes in food availability. For example, when a period of diet restriction ends, animals may increase their growth rate to compensate for the previous lack of resources. This process, named compensatory growth, can help animals to avoid the costs of maturing at a smaller size (Hector and Nakagawa 2012). However, compensatory growth may divert resources away from reproductive and somatic development, leading to long-term fitness consequences (Metcalfe and Monaghan 2001; Mangel and Munch 2005; Dmitriew 2011).

Compensatory growth has been shown to have short- and long-term negative consequences (Mangel and Munch 2005; Hector and Nakagawa 2012; Py et al. 2022). One of the most well-known consequences of compensatory growth is increased exposure to predators (Buskirk and Yurewicz 1998; Lankford et al. 2001; Metcalfe and Monaghan 2003). In order to obtain more resources to sustain higher growth rates, animals usually increase their feeding rates, which in turn can increase their exposure to predators. Another potential cost comes from metabolic changes induced by accelerating growth rates, including hormonal shifts and higher production of reactive oxygen species (Hornick et al. 2000; Janssens and Stoks 2020; Py et al. 2022). This physiological stress during development may affect tissue maturation, leading to long-term detrimental effects (Eyck et al. 2019). Whereas most studies focus on the negative effects on somatic tissues (Hector and Nakagawa 2012; Py et al. 2022), compensatory growth can also affect reproduction. For example, three-spined sticklebacks (*Gasterosteus aculeatus*) expressed nuptial coloration for a shorter duration after compensating for a period of low food availability (Lee et al. 2016). Importantly, if compensatory growth is an adaptive response to short-term food shortage, its long-term costs on reproduction should not outweigh the benefits of maturing at the same size and time as other conspecifics (Metcalfe and Monaghan 2001; Yearsley et al. 2004). This dynamic can become even more complex when reproduction encompasses organs with different repair capacities, including behavior (eg brain), ornaments (eg skin), and sperm (eg testicles; Lindström et al. 2005).

In recent years, increasing attention has been given to how compensatory growth patterns could be modified under new climatic projections. For ectotherms, elevated temperature has a direct effect on metabolism and growth, increasing metabolic demands and the creation of reactive oxygen species. Therefore, higher temperatures could limit resources available for growth, negatively affecting compensatory responses (Lemoine and Burkepile 2012). For example, in the European shore crab (*Carcinus maenas*), compensatory growth is accompanied by high mortality and reduced body mass but only at high temperatures (Torres and Giménez 2020). Additionally, high temperatures usually cause ectotherms to mature earlier and smaller (the “temperature-size rule”), which directly opposes the fitness gains of compensatory growth (Atkinson 1996). Some researchers argue that smaller size can itself be adaptive at higher temperatures, enabling greater reproductive investment in early reproduction (Angilletta and Dunham 2003; Verberk et al. 2021; Wootton et al. 2022). Yet, very few studies have explored the combined effects of fluctuations in food availability and increased temperatures on male reproductive traits. Among those that have, most report that higher temperatures exacerbate negative effects of food availability on reproductive traits (Donelson et al. 2010; Wolnicki et al. 2017; Lopes et al. 2022). For example, convict cichlids exposed to a combination of high temperature and diet restriction showed reduced gonadal development (Abdollahpour et al. 2022). Because higher temperatures have well-known negative effects on size and reproduction (Alix et al. 2020; Dougherty et al. 2024), it is likely that increased temperatures exacerbate existing long-term consequences of compensatory growth.

Trinidadian guppies (*Poecilia reticulata*) are tropical freshwater fish that have been extensively used as a model for studies in ecology and evolution (Houde 1997). They have also been used as an ectothermic model for studies regarding the effects of climate change on animal physiology and reproduction (Imai et al. 2022; Breedveld et al. 2023). Guppies have a complex range of pre- and postcopulatory sexual traits. Males compete with other males to obtain access to females (Price and Helen Rodd 2006; Guerrera et al. 2022). When encountering a female, males either court the female, showing off their body coloration in a sigmoid display, or attempt sneak copulations. The likelihood of performing these mating strategies has been linked to both genetic and environmental factors, including social experience and diet (Kolluru et al. 2007; Evans 2010; Polverino et al. 2019). Female guppies choose mates based on body coloration, often preferring males with relatively larger orange spots with higher saturation (Houde 1997; Brooks and Endler 2001; Venkatesan et al. 2024). Although male coloration in guppies has been linked to genetic factors (Lindholm and Breden 2002; Herdegen et al. 2014), environmental factors (eg food availability and temperature) can also affect body coloration (Devigili et al. 2013; Rahman et al. 2013; Ruell et al. 2013; Breedveld et al. 2023). Additionally, due to their polyandrous mating system, sperm competition is common (Evans and Magurran 2001; Neff et al. 2008; Jeswiet et al. 2011). As a result, male guppies have to adjust investment between pre- and postcopulatory traits across different environments to optimize reproductive fitness (Evans 2010).

Previous empirical research on tropical fish has generally focused on the effects of adult diet or temperature on male sexual traits (food availability: Archard et al. 2006; Kolluru et al. 2007; Devigili et al. 2013; Rahman et al. 2014; Evans et al. 2023; temperature: Breckels and Neff 2013; Breedveld et al. 2023) with little consideration for the effects of environmental conditions experienced during development (but see Evans et al. 2023 and Breckels and Neff 2013). Furthermore, in those cases where diet and temperature have been manipulated during development, they are generally considered in isolation, and so we currently know very little about their potential interactive effects. It is important to address these knowledge gaps because tissue growth and maturation in juvenile fish are highly susceptible to external conditions (McDermott and Safran 2021), and there is growing evidence that compensatory growth early in life can have long-lasting consequences for fish reproduction (Py et al. 2022). In guppies, for instance, females exhibit lower fecundity after experiencing compensatory growth early in life (Auer et al. 2010). It is unclear how increased energy expenditure required for such compensatory growth under high temperatures may affect reproductive traits in male guppies (Ruell et al. 2013; Evans et al. 2023).

Male guppies are an ideal model for studying changes in reproductive traits driven by compensatory growth given their well-studied mating system and the documented environmental effects on sexually selected traits (including diet and temperature; Archard et al. 2006; Kolluru et al. 2007; Devigili et al. 2013; Ruell et al. 2013; Rahman et al. 2014; Breedveld et al. 2023; Evans et al. 2023). Here, we aim to test whether increased temperatures exacerbate the potential long-term costs of compensatory growth in male guppies. To do this, we measure somatic and reproductive traits of adult male guppies that have previously been shown to exhibit compensatory growth in response to an early-life diet restriction (Moura-Campos et al. 2025). Our experimental design involved imposing a 2-wk period of diet restriction on guppies reared at 2 different temperatures (26 °C and 30 °C). Measurement of juvenile growth from this experiment revealed that both male and female guppies show compensatory growth (accelerated growth after diet restriction), irrespective of temperature (Moura-Campos et al. 2025). In the present study, we aim to clarify the potential long-term costs of compensatory growth for male reproduction and whether such costs are exacerbated by increased temperature. To do this, we used the same focal males as those in Moura-Campos et al. (2025), given their observed compensatory growth responses. We measured male pre- and postcopulatory traits, including male–male competition (dominance), male–female interactions (courtship displays and sneak copulation), sperm production, and secondary sexual traits (orange and black coloration) at ∼5 mo of age. Because reproductive traits may trade-off with somatic traits, we also measured body size and relative telomere length (RTL) as proxies for body condition and predicted lifespan at 8 mo of age, although we also monitored mortality throughout adulthood. Telomere shortening indicates cumulative replicative history on chromosomes, which can trigger cell senescence and apoptosis. Telomere length has been widely used as a proxy for somatic aging and predicted lifespan (Olsson et al. 2018). The net outcome of telomere shortening depends on the balance between attrition driven by cell division, oxidative damage, and telomerase-mediated lengthening. A strong telomerase activity in short-lived ectotherms may mask the impact of oxidative stress on telomere shortening (Olsson et al. 2018). However, whether elevated temperatures in combination with compensatory growth modify such telomere dynamics remained unexplored (Friesen et al. 2022).

Data looking at long-term effects of compensatory growth under high temperature in adult females from the same experiment are published elsewhere (Chung et al. 2026). We hypothesized:

1. A negative effect of compensatory growth on male adult traits. At the control temperature, males that experienced diet restriction will invest less in pre- and postcopulatory traits than their well-fed counterparts, leading focal males to court females less and be less dominant (chase opponents less, and be chased more). We also expect to see an increase in the number of attempted sneak copulations, as previous studies have shown that when resource-limited guppies often switch from high energy courtship to low energy sneaking (Devigili et al. 2013). Control-temperature males that experienced diet restriction are also expected to have lower RTL due to increased growth rates (Monaghan and Ozanne 2018). These changes are likely to be primarily driven by compensatory growth, although other physiological consequences of diet restriction may also contribute to negative effects on these traits.
2. A negative effect of high temperatures on male adult traits. For fish without compensatory growth responses (control diet), those at the higher temperature will be smaller (temperature-size rule; Atkinson 1996). We expect them to generally invest less in pre- and postcopulatory traits than those at the control temperature, leading to lower dominance (chase opponents less, and be chased more) and courtship behaviors. We also expect to see an increase in sneak copulations, as described above. We also expect high temperature males to have lower RTL due to increased growth rates exhibited at higher temperatures when compared with control-temperature counterparts (Schulte 2015).
3. A synergistic negative effect of compensatory growth and high temperature on male adult traits. We expect high temperatures to amplify the negative effects of diet restriction (and consequently compensatory growth) because higher metabolic costs at increased temperature may compound the costs of early diet restriction (Huey and Kingsolver 2019). Therefore, fish that experience both diet restriction and high temperature are expected to invest less in pre- and postcopulatory traits than any other treatment group, resulting in the lowest courtship rates and dominance (chase opponents less, and be chased more). We also expect them to attempt more sneak copulations than any other treatment. Due to the synergistic effects of compensatory growth and temperature on their somatic maintenance, we expect them to have the lowest RTL of all treatments.

## Materials and methods

### Fish origin and maintenance

Fish measured in this study came from a large experiment in which we manipulated the temperature and early-life diet of guppies to determine (i) compensatory growth responses under control and high temperature (Moura-Campos et al. 2025), (ii) long-term effects of compensatory growth under the 2 temperatures for female guppy life-history traits (Chung et al. 2026), and (iii) long-term consequences of compensatory growth under the 2 temperatures on male sexually selected traits (this study). Although there is overlap in the fish used in each of these studies [ie juvenile females from (i) are measured as adults in (ii) and juvenile males from (i) are measured as adults in (iii)], there is no overlap in the data presented in each of these papers (Table S1). Details on experimental design are provided in our previous papers (Moura-Campos et al. 2025; Chung et al. 2026) and below.

Guppy stocks used to generate fish for our experiment were descendants from 2 laboratory stocks originally collected from a wild population near Alligator Creek, Townsville, Australia (Lindholm et al. 2014; Kranz et al. 2018). Stock guppies were kept in controlled temperature rooms at 26 ± 1 °C with a 14:10 light/dark cycle, and they were fed twice daily: once with live brine shrimp nauplii (*Artemia* sp.) and once with fish flakes (Aqua One). Stock tanks were inspected weekly, and newborn fry were transferred to 7 L tanks. These juveniles were sexed every week, and when identified as male or female, they were transferred to same-sex tanks to ensure virginity as adults. All animal care and procedures were approved by the ANU Animal Ethics Committee (permit A2021/04).

To generate focal fish for our experiment, we paired a male and a female from virgin stocks in a 7 L plastic tank (*n* = 28 pairs) to breed. After 1 wk together, males were removed from the tank, and we inspected tanks daily for fry. After birth, fry (ie focal individuals) were transferred to individual 1 L treatment tanks where they remained isolated tanks throughout the whole experiment. Each brood was produced by a different female (*n* = 28). Up to 17 fry from each brood were alternately assigned to 1 of the 4 treatments (average 2 fry per brood/treatment) to ensure even representation of each brood across the 4 treatments. We accounted for brood identity in all statistical analyses (see below).

### Early developmental treatments

On the day fry were born, we placed them in their temperature treatment. We manipulated temperature by placing tanks in temperature-controlled rooms at either 26 ± 1 °C (control) or 30 ± 1 °C (high). This equated to water temperatures of 24.4 ± 0.6 °C (control) and 27.8 ± 0.4 °C (high). In their native habitat in Trinidad, guppies experience water temperatures ranging from 20 to 28 °C (Alkins-Koo 2000). Our focal population came from Alligator Creek, Australia, where minimum and maximum (50 yr average) temperatures are 18 and 28.2 °C, respectively (Abatzoglou et al. 2018). Future climate projections predict that average air temperatures will rise by 4 °C by the end of the century (Breckels and Neff 2013; IPCC, 2023). Therefore, our control temperature reflects the current norm temperatures experienced by wild guppies, and our high temperature treatment reflects future temperatures predicted by the IPCC that are likely to be experienced by guppies. Animals were kept in their respective temperature treatment from their day of birth to the end of the experiment.

To manipulate early-life diet, animals were fed ad libitum for 3 d and then placed in their assigned diet treatment (control or restricted). For the following 2 wk, fish on the restricted diet received 3 mg of live brine shrimp (*Artemia* spp.) nauplii every other day, where fish on the nonrestricted diet received 12 mg per day. Because the provided food is fully consumed between feedings, diet-restricted individuals only received one-eighth of the amount offered to nonrestricted counterparts. This restricted feeding regime and quantity has been used effectively in other studies looking at compensatory growth in poeciliid fish (Auer et al. 2010; Vega-Trejo et al. 2016). Importantly, feeding a total of 3 mg of Artemia every other day resulted in almost no growth during the 14-d period of diet restriction (Moura-Campos et al. 2025). After this period of diet restriction, all fish were fed ad libitum, which induced compensatory growth in the diet-restricted group at both temperatures (Moura-Campos et al. 2025). Male guppies take around 50 to 60 d to mature (Houde 1997; Moura-Campos et al. 2025), therefore this 14-d period represents ∼25% of their developmental period. All male guppies were kept in their individual tanks at their original temperature treatment until the end of the experiment.

### Measuring adult male traits

In this study, we aimed to test the long-term consequences of diet restriction/compensatory growth under different temperatures on sexual traits. To do this, we used males from (Moura-Campos et al. 2025) that survived into adulthood. This included a total of 207 male guppies split across 4 different treatments: control diet/control temperature (*n* = 50; 24 broods), restricted diet/control temperature (*n* = 57; 25 broods), control diet/high temperature (*n* = 50; 23 broods), and restricted diet/high temperature (*n* = 50; 22 broods).

Approximately 3 mo after males reached sexual maturity (ie around 5 mo after birth), we carried out all behavioral assays and measured body size, body coloration and sperm traits (see details below). At ∼8 mo of age, we measured their RTL and body size (see details below). We recorded age (number of days) at the first behavioral assay and at the end of the experiment to account for possible age-related effects on sexual and somatic traits (Evans et al. 2002). First behavioral assays were conducted between 111 and 177 d of age. At the end of the experiment, fish age ranged between 194 and 251 d. Due to adult mortality, sample size in the current study varied among traits measured at different times. Details on final sample sizes for each trait are provided in the Supplementary material (Table S2).

### Male–male interaction assays

We conducted behavioral assays to examine male–male interactions in a 56-L aquarium that was kept at the same temperature as the male's original treatment (control/high). To begin a trial, we placed a virgin stock female in the middle of the aquarium, isolated by a 1.5 L transparent glass cylinder (10 cm diameter). We randomly selected a stock male of similar age to our focal individual to act as an intraspecific competitor (hereafter “opponent”). The focal male and a stock male were then placed at opposite ends of the aquarium behind mesh barriers and allowed 10 min to acclimate to the experimental setting. Unique body coloration patterns of each male were used for individual identification. After acclimation, we removed the mesh barrier and started the 10-min observation period. Male guppies display several antagonistic behaviors toward one another, including sexual displays, interferences (one male positioning in front of the other in a rapid movement), nipping, and chasing (Daniel and Williamson 2020; Guerrera et al. 2022). Previous trials with our population showed that the main antagonistic interaction between males was chasing one another (Moura-Campos, personal observation). During observations, we recorded the amount of time the focal male spent chasing their opponent as well as the amount of time the focal male spent being chased by their opponent. We repeated this procedure for all focal males using a new female and opponent male each time. Opponent males were fed ad libitum and were taken from stock tanks kept at the same temperature (for at least a month) as the focal male they were paired with. We kept a total of 50 stock opponent males in 5 tanks at each temperature (ie 10 males per tank). After use, opponent males were returned to a “used male” tank and only once all males had been used did we start selecting males from the used tanks. This meant that stimulus opponent males may have been used up to 3 times but always at least 14 d after their previous trial. Focal males were tested only once. Immediately after the behavioral assay, we anesthetized each opponent using AQUI-S (New Zealand; 20 mg/L) for 30 s and photographed them following standard procedures to allow later measurement of body size (see below). All male–male interaction trials occurred between 1 and 5 PM, and all males were fed 30 min prior to their assay to discourage foraging behavior. All trials were conducted in the same temperature at which the focal fish had been raised since birth and to which the opponent males and stimulus females had acclimated to for 4 wks. During behavioral assays, the observer was blinded to the diet treatment. However, due to the use of different rooms to manipulate temperature, the observer was not blinded to the temperature treatment.

### Male–female interaction assays

We conducted behavioral assays to examine male–female interactions 1 d after the male–male interaction assays. One focal male and 1 virgin female were placed in the aquarium (56 L) and allowed to acclimate for 10 min at opposite ends separated by a mesh. After acclimation, we removed the mesh and observed male–female interactions for 20 min. During this period, we recorded 2 alternative mating strategies of male guppies. Upon encountering a female, males can perform a stereotypical sexual display where they bend their bodies in an “S” shape while swimming up and down (“sigmoid display”; Houde 1997). Alternatively, males can try to surpass female choice by approaching females from behind and thrusting their gonopodium forward to transfer sperm (hereafter “sneak copulation attempt”; Houde 1997). The number of sneak copulation attempts was recorded as the number of gonopodium thrusts toward a female. To ensure their virginity, females were only used once. Both males and females were fed 30 min prior to the assay to discourage foraging behavior. All trials occurred between 9 AM and 12 PM, when animals are most sexually active. All trials were conducted in the same temperature at which the focal fish had been raised since birth and to which the females had acclimated to for 4 wks. As for male–male interaction assays, the observer was blinded to diet treatment but not temperature treatment.

### Male color and size

Immediately after the male–female interaction assays, we anesthetized focal males (AQUI-S, New Zealand; 20 mg/L) for photographing. Each male was gently dried, placed on a white background, and photographed using an Olympus TG-6 digital camera under the same standard conditions illuminated by 2 LED light strips. Each picture contained the male, identification number, a ruler, and a Calibrite colorchecker passport. We analyzed each picture using the software ImageJ to obtain fish standard length (tip of the snout to end of the caudal peduncle), body area, orange spot area, and black spot area. We divided orange and black area by total body area to obtain the proportion of orange and black coloration for each fish. Additionally, we used the micaToolbox bar plugin within the software ImageJ to obtain RGB values for orange spots from RAW pictures (Troscianko and Stevens 2015). From the RGB values, we then calculated mean orange saturation for each orange spot. Further, we calculated the weighted average of orange saturation based on the size of each orange spot, therefore obtaining overall orange saturation values for each fish.

### Sperm number

We collected sperm 3 d after the mating trials to ensure full replenishment (Pilastro et al. 2002). To collect sperm, males were anesthetized, gently dried, and placed on a polyvinyl alcohol-coated slide under a stereomicroscope. We moved their gonopodium forward and gently pressed their ventral region to release the sperm bundles. We temporarily stored the sperm bundles in an extender medium until further analyses (performed less than 1 h after collection). At the start of the analysis, we vortexed the tube for 1 min to break up the sperm bundles and added 3 μL of the solution to a counting slide (Leja Products B.V., the Netherlands). Using the CEROS II software as a Computer Assisted Sperm Analyses system (Hamilton Thorne, United States), we counted the number of sperm cells in 5 areas randomly selected along the slide. The average sperm cell number was then multiplied by the dilution factor to obtain the approximate total sperm number for each fish.

### Body size and RTL

We inspected individual tanks every 3 d throughout the experiment to record mortality. We euthanized all surviving animals at the end of the experiment (*n* = 136). Fish were euthanized by exposure to a lethal overdose of AQUI-S (New Zealand). Immediately after euthanasia, we individually photographed them under standard conditions for later measurement of total body length (as described above). We, therefore, recorded 2 body size measurements: initial body size (mean age: 155 ± 14.9 d) and final body size (mean age 235.3 ± 9.9 d) following the same procedure. We individually stored fish bodies at −80 °C until further processing.

We quantified RTL to examine the effects of temperature and early-life diet on somatic aging. We used only DNA from euthanized fish to ensure adequate tissue preservation. Telomeres are repetitive DNA sequences at the ends of chromosomes that protect the chromosome from deterioration or fusion with neighboring chromosomes (Lansdorp 2005). RTL reflects the cumulative number of cell divisions, and stress a cell has experienced. We used a section of tail muscle (20 mg) to extract genomic DNA using DNeasy Blood and Tissue Kit (QIAGEN). We quantified the concentration and purification of extracted DNA using Qubit dsDNA BR Assay Kits (Thermo Fisher Scientific) and CLARIOstar Plus microplate reader (BMG Labtech), respectively. We used real-time quantitative polymerase chain reaction (qPCR; QuantStudio 3 qPCR system) to determine RTL by measuring how the telomere DNA sample differed from a nonvariable reference DNA sample (melanocortin 1 receptor [MCR1]). We followed the standardized protocol and primers described in recent RTL studies on guppies (Monteforte et al. 2020; Morbiato et al. 2023). We ran each sample, 2 inter-plate calibrator samples and a negative control in triplicate. Standard curves were generated by 4-fold serial dilutions of DNA, which were also run in triplicate. We recorded the cycle number in each qPCR reaction at which the fluorescence signal from amplified DNA exceeded a threshold above background noise (ie *Ct* value). We then used the mean *Ct* value across replicates of a sample to calculate RTL based on a telomere/MCR1 ratio (Monteforte et al. 2020).

### Statistical analyses

We conducted all statistical analyses in R version 4.2.2 (R Core Team 2022). We tested the interactive effects of temperature and early-life diet restriction on 9 traits at ∼5 mo of age: initial size, time spent chasing a male, time spent being chased by a male, number of sneak copulations, number of sigmoid displays, sperm production, black spot area (proportion), orange spot area (proportion), and orange spot saturation. We further measured 2 traits at ∼8 mo of age: final body size and RTL. Finally, we recorded mortality throughout the whole experiment. Thus, in total, we ran individual models for 12 traits.

We fitted linear mixed models (LMMs) using the function “lmer” from the “lme4” package for 6 traits: orange area, black area, orange saturation, initial body size, final body size, and RTL. We visually inspected Q–Q plots to check the normality of the residuals and homogeneity of variance.

Five traits (time chasing, time chased, number of sigmoid displays, number of sneak copulations, and sperm number) did not meet the model assumptions of LMMs, so we analyzed them using generalized LMMs (GLMMs) with negative binomial error family (the function “glmmTMB” from the “glmmTMB” package). For the behavioral traits (time chasing, time chased, number of sigmoid displays, and number of sneak copulations), we first tested for zero-inflation using the function “testZeroInflation” from the “DHARMa” package. The zero-inflation tests were significant for time chased and number of displays, in which, we ran zero-inflated GLMMs that included a zero-inflation part (estimating the likelihood of being zero; binomial error family) and a conditional part (estimating variation in observed events when excluding zero; negative binomial error family). All predictors were included in both parts, resulting in 2 model outputs (occurrence and conditional). Finally, we analyzed mortality using the Cox regression model (“coxme” package) and treated individuals that were alive at the end of the experiment as right-censored data.

For all models, we included brood identity as a random factor and diet and temperature as fixed factors, as well as their interaction. To account for potential size effects on sexual traits, we included initial body size as a covariate in the models of number of sneak copulations, number of displays, and sperm count. For male–male interactions (time spent chasing and time spent being chased), we included the size difference between the focal male and its rival as a covariate because dominance often depends on the relative size of competing males (Harrison et al. 2018; Guerrera et al. 2022). For coloration, we accounted for body size by using the proportion of color spot area relative to total body area. To account for possible age-related effects on sexual and somatic traits (Evans et al. 2002), we included age (number of days) as a covariate in all models.

Statistical summary of fixed effects and covariates in the mixed models was assessed using the function “Anova” from the “car” package, with alpha = 0.05 (2-tailed). If there was a significant interaction between diet and temperature, we conducted Tukey's post hoc pairwise comparisons of model-estimated marginal mean across the 4 treatment groups (using “emmeans” function from the “emmeans” package). Tukey's tests inherently adjust for multiple testing (Nanda et al. 2021). If there was no significant interaction between fixed factors, we removed the interaction term from the final models to allow better interpretation of main effects (Engqvist 2005). Because there is a known negative correlation between investment in coloration and sperm production in male guppies (Evans 2010), we investigated potential trade-offs between these traits by visually inspecting and calculating Pearson correlations between the partial residuals of sperm and all coloration models. A significant negative correlation between the partial residuals under 1 or more of the treatments would indicate a possible trade-off between these traits.

## Results

The full and reduced models, as well as all Tukey's post hoc pairwise comparisons are presented in the Supplementary materials (Tables S3–S16).

### Initial body size

The effect of temperature on initial body size (at 3 mo after maturity, when behavioral assays were conducted) significantly depended on the diet treatment (an interaction: *χ*^2^ = 4.215, *P* = 0.040). Individuals that experienced both diet restriction during early life and a high temperature were smaller than individuals in all other treatments (Table 1; Table S3).

**Table 1:** Raw values (mean ± standard error) and statistical summary showing the effects of fixed factors (diet and temperature) and covariates (age and/or initial body size) using LMMs (for initial body size and coloration) or a GLMM (for sperm count).

Trait		Raw values		Predictor	*χ* ^2^	*P*
**Initial size**						
		Temperature		Temp × diet	4.215	**0**.**040**
		Control (26 °C)	High (30 °C)	Diet	8.802	**0**.**003**
Diet	Control	1.71 ± 0.01	1.68 ± 0.01	Temp	0.011	0.917
	Restricted	1.70 ± 0.01	1.62 ± 0.01	Age	3.206	0.073

**Orange area**						
		Temperature		Temp × diet	0.009	0.926
		Control (26 °C)	High (30 °C)	Diet	1.17	0.279
Diet	Control	0.08 ± 0.01	0.08 ± 0.01	Temp	2.387	0.122
	Restricted	0.09 ± 0.01	0.10 ± 0.01	Age	0.353	0.552

**Orange saturation**						
		Temperature		Temp × diet	0.851	0.356
		Control (26 °C)	High (30 °C)	Diet	0.99	0.320
Diet	Control	0.43 ± 0.01	0.41 ± 0.01	Temp	3.428	0.064

	Restricted	0.46 ± 0.01	0.45 ± 0.01	Age	29.429	**<0.001**
**Black area**						
		Temperature		Temp × diet	0.427	0.514
		Control (26 °C)	High (30 °C)	Diet	0.554	0.457
Diet	Control	0.05 ± 0.006	0.03 ± 0.003	Temp	24.609	**<0.001**
	Restricted	0.06 ± 0.004	0.04 ± 0.004	Age	0.037	0.847

**Sperm number**						
				Temp × diet	0.117	0.733
		Temperature		Diet	3.035	0.081
		Control (26 °C)	High (30 °C)	Temp	0.107	0.743
Diet	Control	2.54 × 10^6^ ± 3.47 × 10^5^	3.24 × 10^6^ ± 5.04 × 10^5^	Body size	7.281	**0**.**007**
	Restricted	3.98 × 10^6^ ± 4.65 × 10^5^	4.36 × 10^6^ ± 6.68 × 10^5^	Age	0.06	0.807

Color traits were presented as the total color spot area divided by total body area to account for body size. Sample sizes for analyses in Table 1 ranged from 138 to 142 males from 27 broods, with 25 to 44 males in each diet-by-temperature group (see Table S2 for full sample size details). Significant *P* values are shown in bold. Full model outputs are provided in Tables S3 and S9–S12.

### Behavioral traits

Behavior during male–male interactions was not influenced by the combined effect of early-life diet and temperature (Table 2). Early-life diet had no main effect on either the time spent chasing or the time spent being chased, but temperature did (Table 2). Focal males reared at the high temperature spent less time chasing and more time being chased by opponent males than those reared at the control temperature (Table 2).

**Table 2:** Raw values (mean ± standard error) and statistical summary showing the effects of fixed factors (diet and temperature) and covariates (age and body size) on behavioral traits using GLMMs.

Trait		Raw values		Predictor	*χ* ^2^	*P*
**Time chasing another male (s)**						
				Temp × diet	1.018	0.313
		Temperature		Diet	0.565	0.452
		Control (26 °C)	High (30 °C)	Temp	13.368	**>0.001**
Diet	Control	59.68 ± 10.28	64.06 ± 18.70	Body size difference	0.098	0.755
	Restricted	100.77 ± 16.09	45.00 ± 12.57	Age	0.284	0.594

**Time being chased by another male (s)**						
*Zero-inflated part*				Temp × diet	0.051	0.821
				Diet	0.927	0.336
				Temp	4.593	**0**.**032**
				Body size difference	0.771	0.380
				Age	0.117	0.732
*Conditional part*				Temp × diet	0.356	0.551
		Temperature		Diet	0.474	0.491
		Control (26 °C)	High (30 °C)	Temp	7.623	**0**.**006**
Diet	Control	35.74 ± 8.52	64.25 ± 10.65	Body size difference	0.071	0.789
	Restricted	34.91 ± 8.13	108.26 ± 15.59	Age	0.224	0.636

**Number of courtship displays**						
*Zero-inflated part*				Temp × diet	0.768	0.381
				Diet	0.048	0.827
				Temp	0.453	0.501
				Body size	0.843	0.359
				Age	0.002	0.965
*Conditional part*				Temp × diet	0.774	0.379
		Temperature		Diet	0.055	0.814
		Control (26 °C)	High (30 °C)	Temp	0.985	0.321
Diet	Control	10.76 ± 1.58	10.97 ± 1.95	Body size	0.915	0.339
	Restricted	9.93 ± 1.06	10.37 ± 1.52	Age	0.048	0.827

**Number of sneak copulation attempts**						
				Temp × diet	1.591	0.207
		Temperature		Diet	0.036	0.849
		Control (26 °C)	High (30 °C)	Temp	0.763	0.382
Diet	Control	1.06 ± 0.30	2.13 ± 0.60	Body size	0.099	0.753
	Restricted	0.93 ± 0.22	1.06 ± 0.20	Age	1.515	0.218

Sample sizes for analyses in Table 2 ranged from 152 to 153 males from 28 broods, with 28 to 44 males in each diet-by-temperature group (see Table S2 for full sample size details). Significant *P* values are shown in bold. Full model outputs are provided in Tables S5–S8.

Neither early-life diet, temperature nor their interaction influenced the likelihood of expressing courtship displays (zero-inflation part) or the number of courtship displays a male performed (conditional part; Table 2). There was also no main effect of early-life diet, temperature, or their interaction on the number of sneak copulation attempts (Table 2).

### Coloration traits

The effect of early-life diet did not depend on the temperature treatment for any coloration traits (Table 1). Males that experienced early-life diet restriction had similar coloration (relative orange spot area, relative black spot area, and orange saturation) compared with males that did not experience a restricted diet (Table 1). Temperature affected relative black spot area but not relative orange spot area or orange saturation (Table 1). Individuals at the high temperature had lower relative black spot area than those at the control temperature.

### Sperm number

There was no effect of early-life diet, temperature, or their interaction on sperm count (Table 2). Larger males produced more sperm (Table 2).

### Trade-offs between coloration traits and sperm number

There was no correlation between sperm number and any coloration traits after controlling for other effects (Figure S1). There were also no correlations between any coloration traits after controlling for other effects (Figure S1).

### Final body size and mortality

The effect of temperature on final body size (8 mo) significantly depended on diet treatment (temperature × diet interaction: *χ*^2^ = 7.482, *P* = 0.006). Individuals that experienced both diet restriction during early life and high temperature were smaller than individuals in all other treatments (Table S14; Fig. 1).

**Figure 1.**
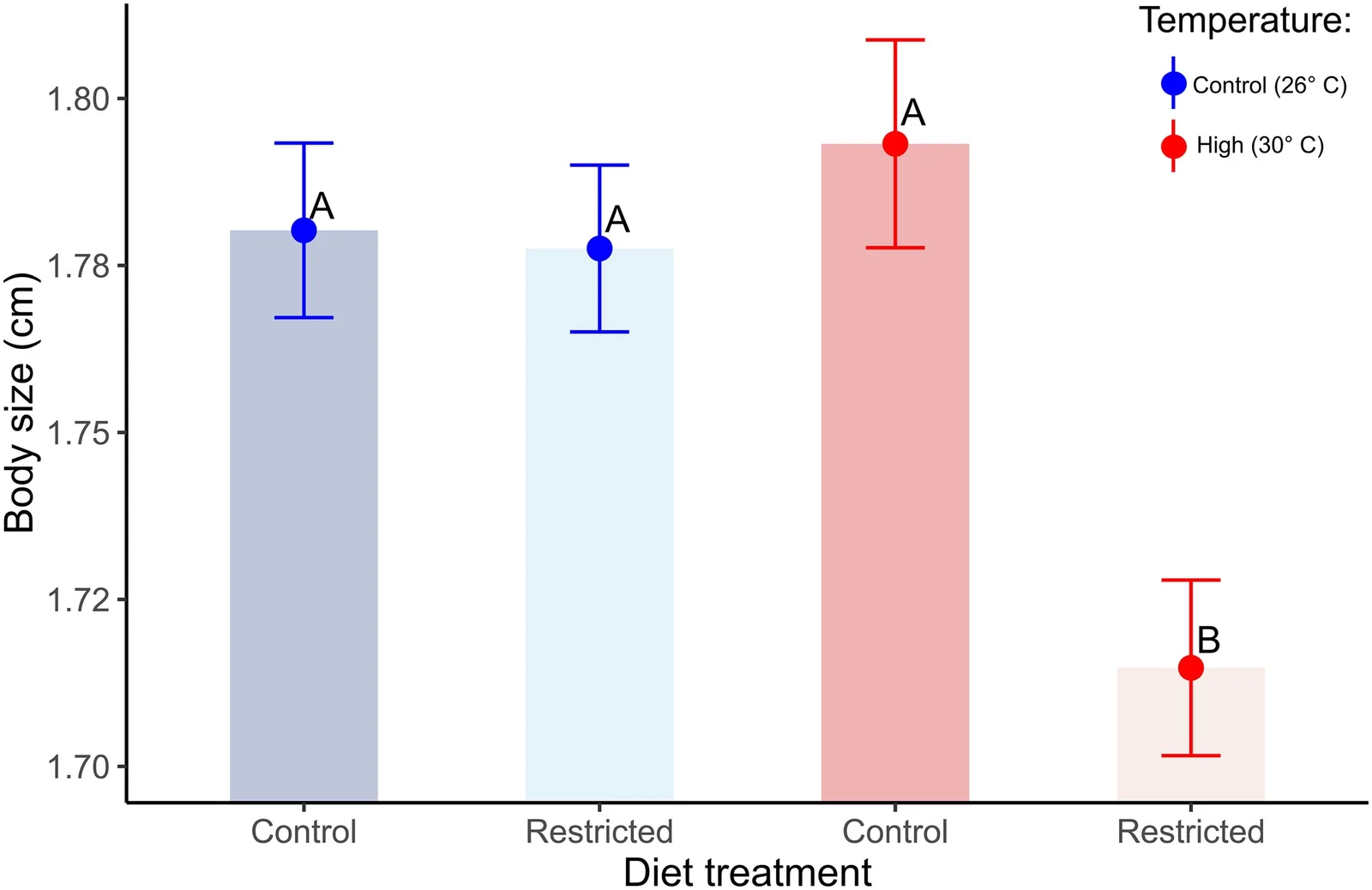
Mean final body size (at eight months of age) separated by treatment (diet * temperature). Dark blue = control diet-control temperature; light blue = restricted diet-control temperature; dark red = control diet-high temperature; light red = restricted diet-high temperature. Error bars are standard errors. Different letters represent significant differences among treatments based on a linear mixed model. Sample size for this analysis consisted of 133 males from 28 broods, with 24 – 41 males in each diet-by-temperature group (see supplementary table 2 for full details). Full model outputs are provided in supplementary tables 13 – 14.

Neither early-life diet (*χ*^2^ = 0.101, *P* = 0.750), temperature (*χ*^2^ = 0.575, *P* = 0.448) nor their interaction (*χ*^2^ = 1.111, *P* = 0.292) significantly affected RTL (Figure S2). Early-life diet and temperature did not interact to affect mortality (*χ*^2^ = 0.023; *P* = 0.881), but they independently affect male mortality (Fig. 2). Males at the high temperature showed a higher mortality rate than those at the control temperature (*χ*^2^ = 7.252; *P* = 0.007), and males that experienced diet restriction early in life had a lower mortality rate than their well-fed counterparts (*χ*^2^ = 4.015, *P* = 0.045).

**Figure 2.**
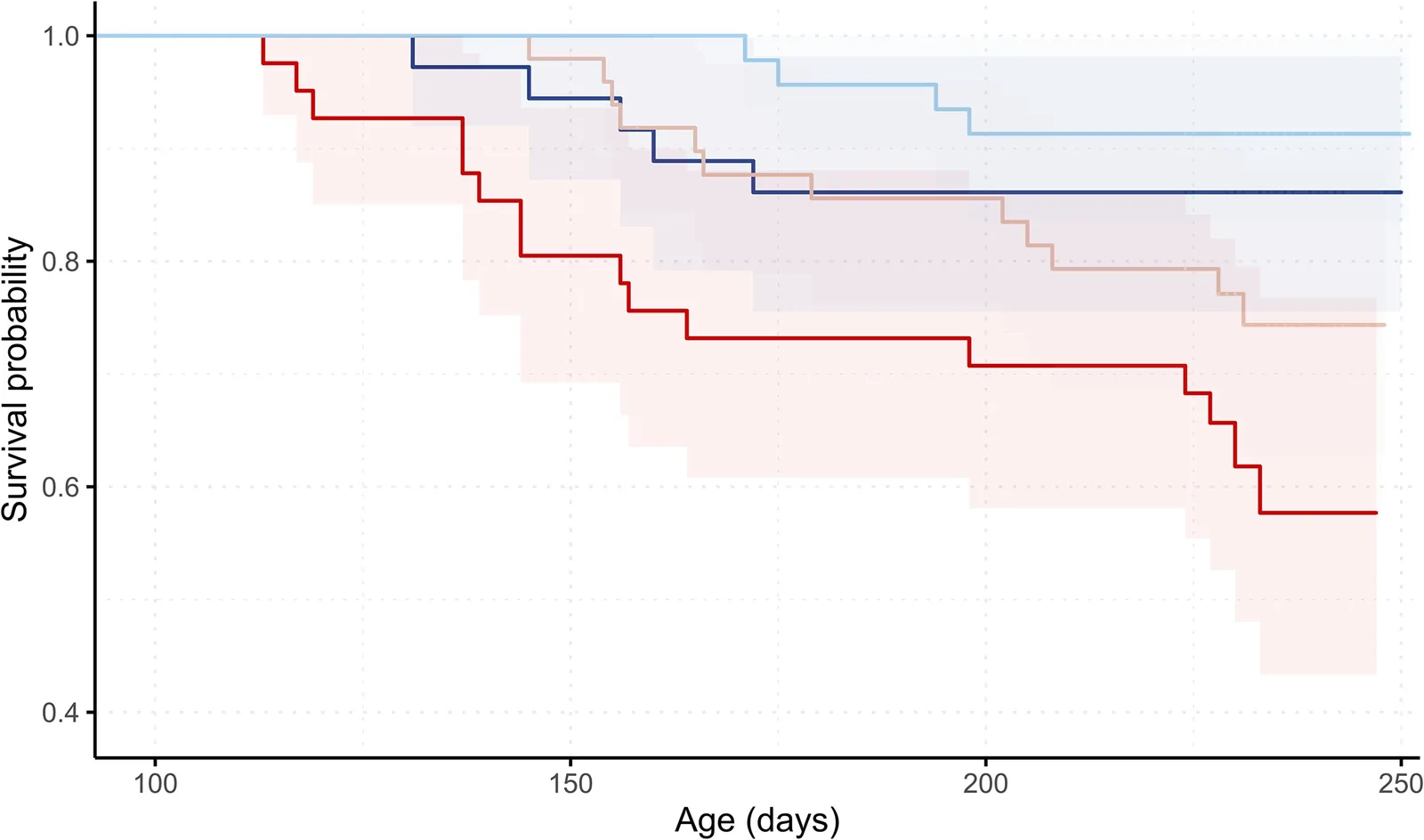
Survival curves based on a Cox regression model of each early-diet and temperature treatment, with corresponding 95% confidence intervals. Line colors represent treatments: control diet-control temperature, restricted diet-control temperature, control diet-high temperature, restricted diet-high temperature. All males were used for mortality analysis, with 50–57 males in each diet-by-temperature group; see Table S2 for full sample size details.

## Discussion

Lack of food can be especially problematic for animals during development because tissue growth is typically maximized during this critical window and requires substantial resource investment (Fawcett and Frankenhuis 2015). To cope with this challenge, many animals have evolved a life-history strategy that allows them to increase their growth rate when food becomes abundant after a period of diet restriction, ie compensatory growth (Dmitriew 2011; Hector and Nakagawa 2012). However, increasing growth early in life can result in trade-offs with traits expressed later in life, ie in a grow now, pay the costs later (Metcalfe and Monaghan 2001). Despite considerable research into compensatory growth and its potential costs (Ali et al. 2003; Mangel and Munch 2005; Hector and Nakagawa 2012; Janssens and Stoks 2020; Py et al. 2022), we know very little about how other environmental factors affect the economics of this strategy. In a previous study, we found that male guppies showed compensatory growth after a period of diet restriction early in life, and that this response was exhibited even under high temperatures (Moura-Campos et al. 2025). Here, using the same focal male fish, we investigated whether compensatory growth imposes long-term reproductive costs and whether such costs are exacerbated by high temperatures. Surprisingly, among 12 measured traits, we only detected an effect of a diet-by-temperature interaction on adult size at both 5 and at 8 mo of age. Contrary to our predictions, there was no main effect of early-life diet restriction (and so presumably compensatory growth) on any sexually selected traits. However, high temperatures alone reduced male dominance, relative area of black coloration, and increased adult mortality. Our findings highlight that compensatory growth does not impose long-term costs on male guppy reproduction, so there was no opportunity for high temperatures to amplify such costs.

### Early-life diet restriction had no effect on male reproductive traits

Contrary to our predictions, we found no effect of early-life diet restriction on the expression of pre- and postcopulatory sexual traits in males. This is surprising because adult diet restriction often reduces investment in male sexual traits (Archard et al. 2006; Kolluru et al. 2007; Devigili et al. 2013; Rahman et al. 2014; Breedveld et al. 2023; Evans et al. 2023). However, most studies manipulated adult diet, thus providing males with few opportunities to compensate for reduced resources. Here, we manipulated diet early in life, and fish went through a period of compensatory growth after diet restriction (Moura-Campos et al. 2025). As a consequence of increased investment in growth, we expected males to reduce investment in sexual traits (ie a trade-off, or cost of compensation), but that was not the case. Interestingly, adult females that underwent compensatory growth during development also showed no reduction in reproductive traits (eg offspring size and number) under the control temperature (Chung et al. 2026), demonstrating that this pattern is consistent across the sexes.

One explanation for our results is that if fish are able to gain enough resources to catch up their growth, they are also able to catch up with other traits (Py et al. 2022). It is possible that guppies are well-adapted to experiencing varying food availability during early development and are therefore able to exhibit compensatory growth without incurring long-term costs. However, we show that early-life diet restriction decreased adult mortality in male guppies. Our focal population from the Alligator Creek is exposed to relatively high levels of predation (Lindholm et al. 2014), which in Trinidad translates to lower population density and higher food availability (Reznick and Travis 2019). However, there is little empirical evidence regarding how this might translate to food availability in our Australian populations. Previous studies manipulating food availability to a similar extent as our study in the same guppies′ population have shown negative effects on sexual traits (Devigili et al. 2013; Rahman et al. 2013; Evans et al. 2023). It is therefore reasonable to predict that diet restriction and compensatory growth would also lead to negative effects later in life. It is also worth noting that not detecting costs of compensatory growth on male sexual traits does not imply that this strategy is not costly. Compensatory growth may impact traits that were not measured in this study and/or impose ecological costs. In nature, an increased growth rate after a period of diet restriction is associated with increased foraging behavior (Ali et al. 2003). If animals forage more after a period of diet restriction, they may be more exposed to predators (Gotthard 2000). Predation could thus be a hidden fitness cost of compensatory growth for both males and females, but such cost cannot be observed under control laboratory conditions. In the absence of predation, diet-restricted guppies might be able to forage effectively once the food is available again and gain sufficient resources for both growth and reproductive investment. Indeed, if fish forage more efficiently after experiencing a period of reduced food availability, this could explain why we found that diet-restricted individuals had lower mortality rates. Future studies could test this hypothesis by investigating the effects of early-life diet restriction on foraging and antipredator behavior.

### High temperature reduced male dominance, black coloration, and increased mortality

Males that experienced the high temperature during development were chased more by their opponent and chased their opponent less. The opposing direction of these effects (ie decrease in chasing and increase in being chased), and the fact that opponent males were acclimated to the temperature at which behavioral assays were conducted, indicates that development in high temperatures decreased dominance of the focal male, rather than the trial temperature itself. Male–male antagonistic interactions allow male guppies to acquire access to females for mating (Kodric-Brown 1993; Price and Helen Rodd 2006). Dominance is often associated with body size and black coloration in male guppies (Santostefano et al. 2019; Guerrera et al. 2022); however, this effect does not appear to be driven by a size difference between focal males and opponent males. Black coloration in guppies is determined by melanin production, which can be decreased by higher temperatures experienced during early development (Zerulla and Stoddard 2021). In vertebrates, there is a known positive correlation between melanin-based coloration and several behavioral syndromes, including aggressiveness (Horth 2003; Ducrest et al. 2008). The observed decrease in dominance could be an outcome of less black coloration. Our results align with growing evidence that elevated temperatures negatively impact fish reproduction (Walsh et al. 2019; Dougherty et al. 2024). Such warming-induced reduction in mating competitiveness can largely determine male reproductive success if short-term temperature fluctuations (eg heatwaves) cause overlapping male cohorts to develop at different temperatures; in contrast, it may not be important if males and their rivals experience similarly elevated temperatures.

Interestingly, under the control diet, males at the high temperature reached the same size later in life as their control-temperature counterparts. This is noteworthy because we previously reported that a high temperature led males to mature earlier and smaller (Moura-Campos et al. 2025). Here, we show that this size effect disappeared later in adulthood despite adults were still at high temperatures. This pattern suggests that the temperature-size rule for male guppies may represent a physiological constraint rather than an adaptive response. Some studies have proposed that the reduction in body size at higher temperatures reflects an adaptive strategy to optimize metabolic efficiency (Angilletta and Dunham 2003; Verberk et al. 2021; Wootton et al. 2022). However, our finding that size differences equilibrate over time indicates that any potentially adaptive response would be lost after maturity. Alternatively, since male size is a sexually selected trait in guppies (Magellan et al. 2005; Guerrera et al. 2022), it could be that the overall physiological benefits of being smaller in high temperatures may not outweigh the fitness advantages of being larger once males start reproducing. We highlight the importance of tracking fitness-related traits throughout an individual's lifetime to explore the adaptive explanation of the temperature-size rule.

### Compensatory growth under high temperatures had few detectable long-term costs

Previously, we have shown that males experiencing diet restriction early in life exhibited compensatory growth even under high temperatures (Moura-Campos et al. 2025). Although effective in the short-term, this accelerated growth is expected to come with costs later in life (Metcalfe and Monaghan 2001; Hector and Nakagawa 2012). Above we discussed how, unexpectedly, a restricted diet early in life led to no long-term consequences for reproductive traits. Importantly, this effect was not mediated by temperature (ie no interaction between early-life diet and temperature), contrasting with our third hypothesis. This finding demonstrates that the costs of compensatory growth on male reproductive traits were not exacerbated by high temperatures. As discussed above, if animals are able to compensate their growth, they may also be able to maintain investment in somatic and reproductive tissues, even under high temperatures. However, it could also be that diet-restricted males at high temperatures invested in reproductive traits at the expense of investing in other traits. In support of this, we found that males which experienced diet restriction under the high temperature never fully caught up in size to males on the control diet even 8 mo later (Fig. 2). Male guppies have low growth rates after maturation, whereby a trade-off between growth and reproduction is expected to occur largely prior to maturity. Here, we highlight that the effects of early-life restricted diet under high temperatures could last throughout adult life and might come with a fitness cost, given that size is also under sexual selection in male guppies (Magellan et al. 2005). Paternity studies could prove an important tool for clarifying the specific contributions of each reproductive trait to fitness and elucidate possible negative consequences under new climatic conditions (Devigili et al. 2015).

### Neither compensatory growth nor high temperature influenced relative telomere length later in life

Despite this persistent effect of multiple stressors on male size, we found no costs of compensatory growth on telomere length, irrespective of temperatures. Poor nutrition and subsequent increased growth rates have been associated with faster aging (Monaghan and Ozanne 2018). However, recent work reveals that telomere degradation can be highly species-specific, in particular, when recovering from stressors experienced during early life (Chatelain et al. 2020). It is surprising that we did not find an effect of the higher temperature on senescence. In ectotherms, high temperature leads to increased metabolism and faster accumulation of oxidative stress, which is linked to faster pace-of-life and shortening of telomeres (Olsson et al. 2018; Friesen et al. 2022). Interestingly, other studies on guppies have also shown no link between aging or stressors (eg predation risk) and telomere shortening (Monteforte et al. 2020; Morbiato et al. 2023). It has been proposed that due to their fast life cycle, guppies might have evolved increased telomerase activity, which would reduce the detectability of telomere shortening caused by oxidative stress (Olsson et al. 2018). Moreover, we expand these findings showing that increased growth rates due to a high temperature, or diet restriction did not lead to any acceleration of male senescence. These results, however, should be interpreted with caution. Our study was conducted under controlled laboratory conditions in the absence of selective pressures present under natural conditions. Changes in reproductive traits may become more detectable under other selective pressures. For example, under higher predation risk, male guppies decreased sexual displays rates and engaged less in antagonistic behaviors against other males (Godin 1995; Kelly and Godin 2001). In addition, we only measured telomere length from individuals that survived until 8 mo of age, which could have created a survival bias in our results. It is possible that the effects of diet or temperature on telomere length were apparent only in more sensitive individuals that died earlier in the experiment. Future research should investigate the effect of compensatory growth under more natural conditions or directly test oxidative stress to understand physiological costs imposed on somatic tissues.

## Conclusion

Climate change is expected to increase global temperatures, which will likely impose physiological constraints on animal development. However, increased temperature may also lead to community-wide changes, such as decreased prey availability (Woodward et al. 2010; Thomas et al. 2017). Understanding how increased temperatures interact with food availability is therefore important for predicting how animals will respond to climate change. We found that temperature did not mediate long-term effects of diet restriction on reproductive traits in male guppies. In contrast, early-life diet restriction did not significantly affect reproductive traits later in life, whereas high temperatures independently decreased male black coloration. Future climate scenarios indicate not only an overall increase in temperatures but also higher variability in temperatures. In our experiment, guppies were reared at high temperatures from birth, allowing fish to acclimate to their rearing environment. It is expected that fluctuating temperatures would be likely to have stronger negative effects than stable temperature, as repeated acclimation itself can be more costly (Muñoz et al. 2012; Ritchie and Friesen 2022). Testing whether temperature variability and heatwaves mediate the effects of compensatory growth following diet restriction on reproduction would thus be a fruitful avenue for future research (Yoneda and Wright 2005; Muñoz et al. 2012; Breedveld et al. 2023).

## Supplementary Material

arag096_Supplementary_Data

## Data Availability

Analyses reported in this article can be reproduced using the data provided by Moura-Campos et al. (2026).
