# Supplementary material for: Developmental plasticity in response to early-life conditions affects male sexual traits in guppies
Source: Behav Ecol. 2026 Aug 19;37(6):arag096. doi: 10.1093/beheco/arag096 (PMC13673275; doi:10.1093/beheco/arag096)
Supplement: Supplementary file 1 [file arag096_supplementary_data.docx]

**Supplementary materials**

for the article titled “Developmental plasticity in response to early-life conditions affects male sexual traits in guppies”

**Supplementary figures**

**Supplementary Figure 1.** Pearson correlation plots of partial residuals between sperm production and colouration traits (black area, orange area and saturation). Lower panels show pairwise scatterplots, diagonal panels show frequency histograms, and upper panels show correlation coefficients across treatments (Corr) and within each treatment group: CDHT (green) = control diet, high temp; CDCT (red) = control diet, control temp; RDHT (purple) = restricted diet, high temp; RDCT (blue) = restricted diet, control temp. Shaded areas indicate 95% confidence intervals. None of the correlations were significant (all P < 0.05).

**Supplementary Figure 2.** Mean relative telomere length separated by treatment (diet * temperature). Bar colours represent treatments: control diet-control temperature (dark blue), restricted diet-control temperature (light blue), control diet-high temperature (dark red), restricted diet-high temperature (light red). Error bars are standard errors. Letters above the mean represent significant differences among treatments.

**Supplementary tables**

**Supplementary Table 1.** Traits analysed on different studies using the same focal fish.

| **Study** | **Journal** | **Sex** | **Size traits** | **Reproductive traits** | **Somatic traits** |
| --- | --- | --- | --- | --- | --- |
| Moura-Campos et al., 2025 | Journal of Animal Ecology | Male/Female | Compensatory growth | - | Age at maturity |
|  |  |  | Growth until maturity |  |  |
|  |  |  | Body size at maturity |  |  |
| Chung et al., 2026 | The American Naturalist | Female | Body size 2w after maturity | Gestation time | Immune response |
|  |  |  | Body size 4w after maturity | Offspring number per brood | Gut length |
|  |  |  | Body size 6w after maturity | Offspring size | Relative Telomere Length |
|  |  |  | Body size 10w after maturity | Overall fecundity | Survival |
| Moura-Campos et al., | this paper | Male | Body size at 5 months | Male dominance (time chasing and being chased) | Relative Telomere Length |
|  |  |  | Body size at 8 months | Number of sexual displays | Survival |
|  |  |  |  | Number of sneak copulations |  |
|  |  |  |  | Orange area colouration |  |
|  |  |  |  | Black area colouration |  |
|  |  |  |  | Orange saturation |  |
|  |  |  |  | Sperm number |  |

**Supplementary Table 2.** Sample size for each trait split by treatment (diet * temp), with number of broods in parentheses.

|  | **Sample size per treatment** | | | |  |
| --- | --- | --- | --- | --- | --- |
|  | Control diet | | Restricted diet | |  |
| **Trait** | Control  temp | High temp | Control temp | High temp | **Total sample size** |
| **Initial size** | 27 (19) | 28 (20) | 44 (24) | 41 (21) | 140 (27) |
| **Chasing opponent** | 34 (22) | 32 (21) | 44 (24) | 43 (22) | 153 (28) |
| **Chased by opponent** | 34 (22) | 32 (21) | 44 (24) | 43 (22) | 153 (28) |
| **Courtship displays** | 34 (22) | 31 (20) | 44 (24) | 43 (22) | 152 (28) |
| **Sneak copulation** | 34 (22) | 31 (20) | 44 (24) | 43 (22) | 152 (28) |
| **Proportion of orange area** | 25 (18) | 28 (20) | 44 (24) | 41 (21) | 138 (27) |
| **Orange area saturation** | 25 (18) | 28 (20) | 44 (24) | 41 (21) | 138 (27) |
| **Proportion of black area** | 27 (19) | 27 (19) | 44 (24) | 41 (21) | 139 (27) |
| **Sperm count** | 31 (20) | 25 (17) | 44 (24) | 42 (22) | 142 (27) |
| **Final size** | 31 (20) | 24 (21) | 41 (24) | 37 (21) | 133 (28) |
| **Relative telomere length** | 29 (18) | 24 (16) | 30 (20) | 30 (18) | 113 (24) |

**Supplementary Table 3.** Results from linear mixed models (LMMs) of body size with chi-square (χ^2^) values for significance tests of estimated parameters for diet, temperature and age.

| Full model | | Estimate | *SE* | *Df* | χ² | *p-value* |
| --- | --- | --- | --- | --- | --- | --- |
| Fixed effects | |  |  |  |  |  |
| Intercept (restricted, control) | | 1.470 | 0.116 | 130.257 | 161.264 | < 0.001 |
| Diet (restricted) | | -0.080 | 0.271 | 126.921 | 8.802 | **0.003** |
| Temperature (control) | | 0.030 | 0.026 | 124.741 | 0.011 | 0.917 |
| Age | | 0.001 | < 0.001 | 129.342 | 3.206 | 0.073 |
| Diet * Temp | | 0.576 | 0.028 | 91.256 | 4.215 | **0.04** |
| Random effect | **Variance** | ***sd*** | **Number of groups** | |  |  |
| Brood ID (intercept) | 0.001 | 0.032 | 28 | |  |  |
| Residual | 0.006 | 0.074 | 28 | |  |  |

**Supplementary Table 4.** Results from estimated marginal means (EMMs) pairwise comparisons among four treatments for initial body size (five months of age) with *t*-ratio values for significance tests.

| Contrast | *t* ratio |  | p-value |
| --- | --- | --- | --- |
| Control diet, control temp - Restricted diet, control temp | 1.064 |  | 0.712 |
| Control diet, control temp - Control diet, high temp | -0.104 |  | 0.999 |
| Control diet, control temp - Restricted diet, high temp | -4.186 |  | **< 0.001** |
| Restricted diet, control temp - Control diet, high temp | 0.629 |  | 0.922 |
| Restricted diet, control temp - Restricted diet, high temp | -3.391 |  | **0.005** |
| Control diet, high temp - Restricted diet, high temp | 2.942 |  | **0.02** |

**Supplementary Table 5.** Results from generalized linear mixed models (GLMMs) of time chasing another male with chi-square (χ^2^) values for significance tests of estimated parameters for diet, temperature, body size difference between males and age.

| Full model | | Estimate | *SE* | *Df* | χ² | *p-value* |
| --- | --- | --- | --- | --- | --- | --- |
| Fixed effects | |  |  |  |  |  |
| Intercept (restricted, control) | | 4.176 | 1.618 | 1 | 6.663 | 0.010 |
| Diet (restricted) | | -0.110 | 0.399 | 1 | 0.077 | 0.782 |
| Temperature (control) | | 0.552 | 0.358 | 1 | 2.385 | 0.122 |
| Body size | | 0.156 | 0.801 | 1 | 0.038 | 0.845 |
| Age | | -0.002 | 0.012 | 1 | 0.044 | 0.833 |
| Diet * Temp | | 0.393 | 0.389 | 1 | 1.018 | 0.313 |
| Random effect | **Variance** | ***sd*** | **Number of groups** | |  |  |
| Brood ID (intercept) | < 0.001 | < 0.001 | 28 | |  |  |
|  |  |  |  | |  |  |
| Reduced model | | **Estimate** | ***SE*** | ***Df*** | **χ²** | ***p-value*** |
| Fixed effects | |  |  |  |  |  |
| Intercept (restricted, control) | | 4.515 | 1.597 | 1 | 7.992 | 0.005 |
| Diet (restricted) | | 0.197 | 0.262 | 1 | 0.565 | 0.452 |
| Temperature (control) | | 0.835 | 0.228 | 1 | 13.368 | **< 0.001** |
| Body size difference | | 0.248 | 0.794 | 1 | 0.098 | 0.755 |
| Age | | -0.006 | 0.011 | 1 | 0.284 | 0.594 |
| Random effect | **Variance** | ***sd*** | **Number of groups** | |  |  |
| Brood ID (intercept) | < 0.001 | < 0.001 | 28 | |  |  |

**Supplementary Table 6.** Results from generalised linear mixed models (GLMMs) of with chi-square (χ^2^) values for significance tests of time being chased by another male estimated parameters for diet, temperature, body size difference between males and age.

(A) Zero-inflation part

| Full zero-inflated model | | Estimate | *SE* | *Df* | χ² | *P* |
| --- | --- | --- | --- | --- | --- | --- |
| Fixed effects | |  |  |  |  |  |
| Intercept (restricted, control) | | -2.550 | 13.974 | 1 | 0.033 | 0.855 |
| Diet (restricted) | | 2.910 | 8.676 | 1 | 0.113 | 0.737 |
| Temperature (control) | | 3.996 | 8.606 | 1 | 0.216 | 0.642 |
| Body size | | 3.171 | 3.495 | 1 | 0.823 | 0.364 |
| Age | | -0.022 | 0.062 | 1 | 0.123 | 0.726 |
| Diet * Temp | | -1.969 | 8.697 | 1 | 0.051 | 0.821 |
| Random effect | **Variance** | ***sd*** | **Number of groups** | |  |  |
| Brood ID (intercept) | 0.861 | 0.928 | 28 | |  |  |
|  |  |  |  | |  |  |
| Reduced zero-inflated model | | **Estimate** | ***SE*** | ***Df*** | **χ²** | ***P*** |
| Fixed effects | |  |  |  |  |  |
| Intercept (restricted, control) | | -1.297 | 8.114 | 1 | 0.026 | 0.873 |
| Diet (restricted) | | 1.034 | 1.074 | 1 | 0.927 | 0.336 |
| Temperature (control) | | 2.240 | 1.045 | 1 | 4.593 | **0.032** |
| Body size difference | | 2.858 | 3.255 | 1 | 0.771 | 0.380 |
| Age | | -0.019 | 0.056 | 1 | 0.117 | 0.732 |
| Random effect | **Variance** | ***sd*** | **Number of groups** | |  |  |
| Brood ID (intercept) | 0.939 | 0.969 | 28 | |  |  |

(B) Condition part

| Full conditional model | | Estimate | *SE* | *Df* | χ² | *p-value* |
| --- | --- | --- | --- | --- | --- | --- |
| Fixed effects | |  |  |  |  |  |
| Intercept (restricted, control) | | 3.714 | 2.060 | 1 | 3.252 | 0.071 |
| Diet (restricted) | | 0.464 | 0.507 | 1 | 0.838 | 0.360 |
| Temperature (control) | | -0.564 | 0.461 | 1 | 1.494 | 0.222 |
| Body size | | -0.120 | 1.025 | 1 | 0.014 | 0.907 |
| Age | | 0.004 | 0.015 | 1 | 0.057 | 0.812 |
| Diet * Temp | | -0.309 | 0.517 | 1 | 0.356 | 0.551 |
| Random effect | **Variance** | ***sd*** | **Number of groups** | |  |  |
| Brood ID (intercept) | < 0.001 | < 0.001 | 28 | |  |  |
|  |  |  |  | |  |  |
| Reduced conditional model | | **Estimate** | ***SE*** | ***Df*** | **χ²** | ***p-value*** |
| Fixed effects | |  |  |  |  |  |
| Intercept (restricted, control) | | 3.403 | 1.971 | 1 | 2.980 | 0.084 |
| Diet (restricted) | | 0.247 | 0.359 | 1 | 0.474 | 0.491 |
| Temperature (control) | | -0.782 | 0.283 | 1 | 7.623 | **0.006** |
| Body size | | -0.265 | 0.993 | 1 | 0.071 | 0.789 |
| Age | | 0.007 | 0.014 | 1 | 0.224 | 0.636 |
| Random effect | **Variance** | ***sd*** | **Number of groups** | |  |  |
| Brood ID (intercept) | < 0.001 | < 0.001 | 28 | |  |  |

**Supplementary Table 7.** Results from generalised linear mixed models (GLMMs) of number of sigmoid displays with chi-square (χ^2^) values for significance tests of estimated parameters for diet, temperature, body size and age.

(A) Zero-inflation part

| Full zero-inflated model | | Estimate | *SE* | *Df* | χ² | *p-value* |
| --- | --- | --- | --- | --- | --- | --- |
| Fixed effects | |  |  |  |  |  |
| Intercept (restricted, control) | | 3.676 | 6.767 | 1 | 0.295 | 0.587 |
| Diet (restricted) | | 0.515 | 1.245 | 1 | 0.171 | 0.679 |
| Temperature (control) | | 0.303 | 1.172 | 1 | 0.067 | 0.796 |
| Body size | | -2.475 | 3.562 | 1 | 0.483 | 0.487 |
| Age | | -0.011 | 0.036 | 1 | 0.088 | 0.766 |
| Diet * Temp | | -1.200 | 1.370 | 1 | 0.768 | 0.381 |
| Random effect | **Variance** | ***sd*** | **Number of groups** | |  |  |
| Brood ID (intercept) | < 0.001 | < 0.001 | 28 | |  |  |
| Reduced zero-inflated model | | **Estimate** | ***SE*** | ***Df*** | **χ²** | ***p-value*** |
| Fixed effects | |  |  |  |  |  |
| Intercept (restricted, control) | | 3.363 | 6.684 | 1 | 0.253 | 0.615 |
| Diet (restricted) | | -0.187 | 0.855 | 1 | 0.048 | 0.827 |
| Temperature (control) | | -0.495 | 0.735 | 1 | 0.453 | 0.501 |
| Body size difference | | -3.125 | 3.404 | 1 | 0.843 | 0.359 |
| Age | | 0.001 | 0.031 | 1 | 0.002 | 0.965 |
| Random effect | **Variance** | ***sd*** | **Number of groups** | |  |  |
| Brood ID (intercept) | < 0.001 | < 0.001 | 28 | |  |  |

(B) Condition part

| Full conditional model | | Estimate | *SE* | *Df* | χ² | *p-value* |
| --- | --- | --- | --- | --- | --- | --- |
| Fixed effects | |  |  |  |  |  |
| Intercept (restricted, control) | | 1.675 | 1.785 | 1 | 0.881 | 0.348 |
| Diet (restricted) | | 0.180 | 0.347 | 1 | 0.270 | 0.603 |
| Temperature (control) | | 0.043 | 0.308 | 1 | 0.020 | 0.888 |
| Body size | | 1.038 | 0.910 | 1 | 1.303 | 0.254 |
| Age | | -0.006 | 0.010 | 1 | 0.378 | 0.539 |
| Diet * Temp | | -0.300 | 0.341 | 1 | 0.774 | 0.379 |
| Random effect | **Variance** | ***sd*** | **Number of groups** | |  |  |
| Brood ID (intercept) | < 0.001 | < 0.001 | 28 | |  |  |
| Reduced conditional model | | **Estimate** | ***SE*** | ***Df*** | **χ²** | ***p-value*** |
| Fixed effects | |  |  |  |  |  |
| Intercept (restricted, control) | | 1.503 | 1.791 | 1 | 0.704 | 0.402 |
| Diet (restricted) | | -0.052 | 0.223 | 1 | 0.055 | 0.814 |
| Temperature (control) | | -0.179 | 0.180 | 1 | 0.985 | 0.321 |
| Body size | | 0.849 | 0.888 | 1 | 0.915 | 0.339 |
| Age | | -0.002 | 0.009 | 1 | 0.048 | 0.827 |
| Random effect | **Variance** | ***sd*** | **Number of groups** | |  |  |
| Brood ID (intercept) | < 0.001 | < 0.001 | 28 | |  |  |

**Supplementary Table 8.** Results from generalized linear mixed models (GLMMs) of number of sneak copulations attempts with chi-square (χ^2^) values for significance tests of estimated parameters for diet, temperature, body size and age.

| Full model | | Estimate | *SE* | *Df* | χ² | *p-value* |
| --- | --- | --- | --- | --- | --- | --- |
| Fixed effects | |  |  |  |  |  |
| Intercept (restricted, control) | | 3.433 | 3.246 | 1 | 1.118 | 0.290 |
| Diet (restricted) | | -0.467 | 0.587 | 1 | 0.633 | 0.426 |
| Temperature (control) | | -0.840 | 0.549 | 1 | 2.337 | 0.126 |
| Body size | | -0.936 | 1.745 | 1 | 0.288 | 0.592 |
| Age | | -0.009 | 0.016 | 1 | 0.297 | 0.586 |
| Diet * Temp | | 0.779 | 0.618 | 1 | 1.591 | 0.207 |
| Random effect | **Variance** | ***sd*** | **Number of groups** | |  |  |
| Brood ID (intercept) | 0.155 | 0.393 | 28 | |  |  |
| Reduced model | | **Estimate** | ***SE*** | ***Df*** | **χ²** | ***p-value*** |
| Fixed effects | |  |  |  |  |  |
| Intercept (restricted, control) | | 3.836 | 3.254 | 1 | 1.390 | 0.238 |
| Diet (restricted) | | 0.076 | 0.398 | 1 | 0.036 | 0.849 |
| Temperature (control) | | -0.283 | 0.324 | 1 | 0.763 | 0.382 |
| Body size | | -0.545 | 1.730 | 1 | 0.099 | 0.753 |
| Age | | -0.018 | 0.014 | 1 | 1.515 | 0.218 |
| Random effect | **Variance** | ***sd*** | **Number of groups** | |  |  |
| Brood ID (intercept) | 0.188 | 0.437 | 28 | |  |  |

**Supplementary Table 9.** Results from linear mixed models (LMMs) of sperm production with chi-square (χ^2^) values for significance tests of estimated parameters for diet, temperature, body size and age.

| Full model | | Estimate | *SE* | *Df* | χ² | *p-value* |
| --- | --- | --- | --- | --- | --- | --- |
| Fixed effects | |  |  |  |  |  |
| Intercept (restricted, control) | | -10.370 | 27.333 | 1 | 0.144 | 0.704 |
| Diet (restricted) | | 4.324 | 4.806 | 1 | 0.809 | 0.368 |
| Temperature (control) | | -2.048 | 4.363 | 1 | 0.220 | 0.639 |
| Body size | | 34.984 | 13.608 | 1 | 6.609 | 0.010 |
| Age | | -0.015 | 0.143 | 1 | 0.011 | 0.917 |
| Diet * Temp | | 1.610 | 4.710 | 1 | 0.117 | 0.733 |
| Random effect | **Variance** | ***sd*** | **Number of groups** | |  |  |
| Brood ID (intercept) | 18.76 | 4.331 | 28 | |  |  |
| Residual | 135.18 | 11.627 | 28 | |  |  |
| Reduced model | | **Estimate** | ***SE*** | ***Df*** | **χ²** | ***p-value*** |
| Fixed effects | |  |  |  |  |  |
| Intercept (restricted, control) | | -9.976 | 27.214 | 1 | 0.134 | 0.714 |
| Diet (restricted) | | 5.549 | 3.185 | 1 | 3.035 | 0.081 |
| Temperature (control) | | -0.861 | 2.626 | 1 | 0.107 | 0.743 |
| Body size | | 35.877 | 13.296 | 1 | 7.281 | **0.007** |
| Age | | -0.032 | 0.132 | 1 | 0.060 | 0.807 |
| Random effect | **Variance** | ***sd*** | **Number of groups** | |  |  |
| Brood ID (intercept) | 18.48 | 4.299 | 28 | |  |  |
| Residual | 134.32 | 11.590 | 28 | |  |  |

**Supplementary Table 10.** Results from linear mixed models (LMMs) of proportion of black spot area with chi-square (χ^2^) values for significance tests of estimated parameters for diet, temperature and age.

| Full model | | Estimate | *SE* | *Df* | χ² | *p-value* |
| --- | --- | --- | --- | --- | --- | --- |
| Fixed effects | |  |  |  |  |  |
| Intercept (restricted, control) | | 0.690 | 0.049 | 1 | 196.792 | <0.001 |
| Diet (restricted) | | 0.001 | 0.012 | 1 | 0.004 | 0.947 |
| Temperature (control) | | 0.028 | 0.011 | 1 | 6.377 | 0.012 |
| Age | | <0.001 | <0.001 | 1 | 0.150 | 0.698 |
| Diet * Temp | | 0.008 | 0.012 | 1 | 0.427 | 0.514 |
| Random effect | **Variance** | ***sd*** | **Number of groups** | |  |  |
| Brood ID (intercept) | < 0.001 | 0.009 | 28 | |  |  |
| Residual | < 0.001 | 0.328 | 28 | |  |  |
| Reduced model | | **Estimate** | ***SE*** | ***Df*** | **χ²** | ***p-value*** |
| Fixed effects | |  |  |  |  |  |
| Intercept (restricted, control) | | 0.698 | 0.048 | 1 | 214.759 | < 0.001 |
| Diet (restricted) | | 0.006 | 0.008 | 1 | 0.554 | 0.457 |
| Temperature (control) | | 0.034 | 0.007 | 1 | 24.609 | < 0.001 |
| Age | | <0.001 | <0.001 | 1 | 0.037 | 0.847 |
| Random effect | **Variance** | ***sd*** | **Number of groups** | |  |  |
| Brood ID (intercept) | < 0.001 | 0.009 | 28 | |  |  |
| Residual | 0.001 | 0.322 | 28 | |  |  |

**Supplementary Table 11.** Results from linear mixed models (LMMs) of proportion of orange spot area with chi-square (χ^2^) values for significance tests of estimated parameters for diet, temperature and age.

| Full model | | Estimate | *SE* | *Df* | χ² | *p-value* |
| --- | --- | --- | --- | --- | --- | --- |
| Fixed effects | |  |  |  |  |  |
| Intercept (restricted, control) | | 0.059 | 0.044 | 1 | 1.844 | 0.174 |
| Diet (restricted) | | 0.007 | 0.010 | 1 | 0.504 | 0.478 |
| Temperature (control) | | -0.010 | 0.010 | 1 | 1.028 | 0.311 |
| Age | | < 0.001 | < 0.001 | 1 | 0.349 | 0.555 |
| Diet * Temp | | 0.001 | 0.011 | 1 | 0.009 | 0.926 |
| Random effect | **Variance** | ***sd*** | **Number of groups** | |  |  |
| Brood ID (intercept) | < 0.001 | 0.019 | 28 | |  |  |
| Residual | < 0.001 | 0.027 | 28 | |  |  |
| Reduced model | | **Estimate** | ***SE*** | ***Df*** | **χ²** | ***p-value*** |
| Fixed effects | |  |  |  |  |  |
| Intercept (restricted, control) | | 0.060 | 0.042 | 1 | 2.050 | 0.152 |
| Diet (restricted) | | 0.008 | 0.007 | 1 | 1.170 | 0.279 |
| Temperature (control) | | -0.009 | 0.006 | 1 | 2.387 | 0.122 |
| Age | | < 0.001 | < 0.001 | 1 | 0.353 | 0.552 |
| Random effect | **Variance** | ***sd*** | **Number of groups** | |  |  |
| Brood ID (intercept) | < 0.001 | 0.019 | 28 | |  |  |
| Residual | < 0.001 | 0.271 | 28 | |  |  |

**Supplementary Table 12.** Results from linear mixed models (LMMs) of orange spot area saturation with chi-square (χ^2^) values for significance tests of estimated parameters for diet, temperature and age.

| Full model | | Estimate | *SE* | *Df* | χ² | *p-value* |
| --- | --- | --- | --- | --- | --- | --- |
| Fixed effects | |  |  |  |  |  |
| Intercept (restricted, control) | | 0.036 | 0.070 | 1 | 0.270 | 0.603 |
| Diet (restricted) | | -0.022 | 0.016 | 1 | 1.861 | 0.173 |
| Temperature (control) | | -0.029 | 0.016 | 1 | 3.470 | 0.062 |
| Age | | 0.003 | 0.001 | 1 | 29.620 | < 0.001 |
| Diet * Temp | | 0.016 | 0.017 | 1 | 0.851 | 0.356 |
| Random effect | **Variance** | ***sd*** | **Number of groups** | |  |  |
| Brood ID (intercept) | < 0.001 | 0.217 | 28 | |  |  |
| Residual | 0.002 | 0.044 | 28 | |  |  |
| Reduced model | | **Estimate** | ***SE*** | ***Df*** | **χ²** | ***p-value*** |
| Fixed effects | |  |  |  |  |  |
| Intercept (restricted, control) | | 0.053 | 0.067 | 1 | 0.608 | 0.436 |
| Diet (restricted) | | -0.012 | 0.012 | 1 | 0.990 | 0.320 |
| Temperature (control) | | -0.018 | 0.010 | 1 | 3.428 | 0.064 |
| Age | | 0.003 | <0.001 | 1 | 29.429 | **< 0.001** |
| Random effect | **Variance** | ***sd*** | **Number of groups** | |  |  |
| Brood ID (intercept) | < 0.001 | 0.022 | 28 | |  |  |
| Residual | 0.002 | 0.044 | 28 | |  |  |

**Supplementary Table 13.** Results from linear mixed models (LMMs) of body size at eight months with chi-square (χ^2^) values for significance tests of estimated parameters for diet, temperature and age.

| Full model | | Estimate | *SE* | *Df* | χ² | *p-value* |
| --- | --- | --- | --- | --- | --- | --- |
| Fixed effects | |  |  |  |  |  |
| Intercept (restricted, control) | | 1.690 | 0.162 | 120.600 | 109.391 | < 0.001 |
| Diet (restricted) | | -0.078 | 0.020 | 122.700 | 15.221 | **< 0.001** |
| Temperature (control) | | -0.011 | 0.021 | 124.600 | 0.270 | 0.603 |
| Age | | < 0.001 | 0.001 | 120.900 | 0.397 | 0.528 |
| Diet * Temp | | 0.074 | 0.027 | 117.800 | 7.482 | **0.006** |
| Random effect | **Variance** | ***sd*** | **Number of groups** | |  |  |
| Brood ID (intercept) | < 0.001 | 0.022 | 28 | |  |  |

**Supplementary Table 14.** Results from estimated marginal means (EMMs) pairwise comparisons among four treatments for body size at eight months with t-ratio values for significance tests.

| Contrast | t ratio |  | p-value |
| --- | --- | --- | --- |
| Control diet, control temp - Restricted diet, control temp | 0.514 |  | 0.955 |
| Control diet, control temp - Control diet, high temp | 0.247 |  | 0.994 |
| Control diet, control temp - Restricted diet, high temp | -3.558 |  | **0.003** |
| Restricted diet, control temp - Control diet, high temp | 0.770 |  | 0.868 |
| Restricted diet, control temp - Restricted diet, high temp | -3.609 |  | **0.002** |
| Control diet, high temp - Restricted diet, high temp | 3.871 |  | **0.001** |

**Supplementary Table 15.** Results from linear mixed models (LMMs) of relative telomere length with chi-square (χ^2^) values for significance tests of estimated parameters for diet, temperature and age.

| Full model | | Estimate | *SE* | *Df* | χ² | *p-value* |
| --- | --- | --- | --- | --- | --- | --- |
| Fixed effects | |  |  |  |  |  |
| Intercept (restricted, control) | | 0.767 | 1.007 | 1 | 0.580 | 0.446 |
| Diet (restricted) | | -0.062 | 0.063 | 1 | 0.979 | 0.322 |
| Temperature (control) | | -0.017 | 0.065 | 1 | 0.069 | 0.792 |
| Age | | 0.001 | 0.004 | 1 | 0.055 | 0.815 |
| Diet * Temp | | 0.091 | 0.086 | 1 | 1.111 | 0.292 |
| Random effect | **Variance** | ***sd*** | **Number of groups** | |  |  |
| Brood ID (intercept) | 0.043 | 0.208 | 28 | |  |  |
| Residual | 0.046 | 0.215 | 28 | |  |  |
| Reduced model | | **Estimate** | ***SE*** | ***Df*** | **χ²** | ***p-value*** |
| Fixed effects | |  |  |  |  |  |
| Intercept (restricted, control) | | 0.822 | 1.007 | 1 | 0.667 | 0.414 |
| Diet (restricted) | | -0.014 | 0.043 | 1 | 0.101 | 0.750 |
| Temperature (control) | | 0.033 | 0.044 | 1 | 0.575 | 0.448 |
| Age | | 0.001 | 0.004 | 1 | 0.023 | 0.879 |
| Random effect | **Variance** | ***sd*** | **Number of groups** | |  |  |
| Brood ID (intercept) | 0.044 | 0.209 | 28 | |  |  |
| Residual | 0.462 | 0.215 | 28 | |  |  |

**Supplementary Table 16.** Results from Cox mixed models of mortality with values for significance tests of estimated parameters for diet and temperature.

| Full model | | Estimate | *SE* | *z* | *p-value* |
| --- | --- | --- | --- | --- | --- |
| Fixed effects | |  |  |  |  |
| Temperature (control) | | -1.121 | 0.533 | -2.100 | 0.035 |
| Diet (restricted) | | -0.706 | 0.389 | -1.820 | 0.069 |
| Diet * Temp | | 0.118 | 0.783 | 0.150 | 0.881 |
| Random effect | **Variance** | ***sd*** | **Number of groups** | |  |
| Brood ID (intercept) | 0.486 | 0.697 | 28 | |  |
| Reduced model | | **Estimate** | ***SE*** | ***z*** | ***p-value*** |
| Fixed effects | |  |  |  |  |
| Temperature (control) | | -1.069 | 0.397 | -2.690 | **0.007** |
| Diet (restricted) | | -0.677 | 0.338 | -2.000 | **0.045** |
| Random effect | **Variance** | ***sd*** | **Number of groups** | |  |
| Brood ID (intercept) | 0.485 | 0.696 | 28 | |  |
